# Halogen bonding as a supramolecular dynamics catalyst

**DOI:** 10.1038/s41467-019-08878-8

**Published:** 2019-02-22

**Authors:** Patrick M. J. Szell, Scott Zablotny, David L. Bryce

**Affiliations:** Department of Chemistry and Biomolecular Sciences & Centre for Catalysis Research and Innovation, University of Ottawa, Ottawa, ON K1N 6N5 Canada

## Abstract

Dynamic processes have many implications in functional molecules, including catalysts, enzymes, host-guest complexes, and molecular machines. Here, we demonstrate via deuterium NMR relaxation experiments how halogen bonding directly impacts the dynamics in solid 2,3,5,6-tetramethylpyrazine cocrystals, catalyzing the methyl group rotation. On average, we observe a reduction of 56% in the rotational activation energy of the methyl groups in the halogen bonded cocrystals, contrasting the reduction of 36% in the hydrogen bonded cocrystals, with respect to pure 2,3,5,6-tetramethylpyrazine. Density functional theory calculations attribute this superior catalytic ability of the halogen bond to the simultaneous destabilization of the staggered conformation and stabilization of the gauche conformation, overall reducing the rotational energy barrier. Furthermore, the calculations suggest that the catalytic ability of the halogen bond may be tuneable, with stronger halogen bond donors acting as superior dynamics catalysts. Thus, halogen bonding may play a role in both assembly and promoting dynamical processes.

## Abstract (web summary)

The halogen bond is well known for its ability to assemble supramolecules. Here, using NMR experiments, the authors reveal the role of these bonds in dynamic processes, finding that the halogen bond directly catalyzes dynamical rotation in solid cocrystals by reducing the associated energy barrier.

## Introduction

The world of molecules and solids is far from static. Indeed, molecules exhibit well-known degrees of freedom, from simple rotations to more complex movements, which have been applied in the design of effective rotating catalysts^1^, molecular gyroscopes^2^, and to understand how guests, such as CO_2_, behave once adsorbed in a host^3,4^. Dynamics also play pivotal roles in much larger systems, such as biological macromolecules^5^, with faulty dynamical processes within enzymes being associated with disease^6^. Fortunately, these complex movements can be exploited, with molecular machines^7^ recently having risen in prominence, yielding the 2016 Nobel Prize in Chemistry^8^, with several configurations relying on rotations^9–11^, such as molecular motors^12,13^ and propellers^14^. However, the impact of introducing interacting moieties on such dynamical processes remains unclear, and the rapid rotations of methyl groups offer an opportunity to gain insights into how molecular rotations can be affected by changes in the local chemical environment.

There have been sparse reports on how van der Waals interactions, such as those associated with inter- or intramolecular contacts, can affect the energy barrier of a methyl group’s rotation^15–18^. Counterintuitively, both increases and decreases in the rotational energy barrier have been observed upon the introduction of van der Waals contacts, rather than the perhaps expected consistent increase due to steric hindrance. Examples of decreases in the rotational energy barrier have been rationalized as resulting from the destabilization of the staggered conformation of the rotator, overall reducing the barrier^15^. This effect from van der Waals contacts on a methyl group has been observed in a select few instances in polymers^19^ and proteins^20^. In the case of proteins, increases and decreases in the methyl rotational energy barrier have both been observed, and the presence of ligands has been shown to play a critical role in their internal dynamics^21^. While these effects are structurally localized, they potentially could be exploited in the design of ligands and molecular machines through the incorporation of functional groups associated with a potent dynamical catalytic ability.

Halogen bonding (RX^…^Y; X = I, Br, Cl, F; Y = electron donor) is a non-covalent interaction akin to the hydrogen bond^22^ which is currently experiencing a surge of interest in supramolecular chemistry^23,24^, and related fields due to its directionality^25^ and tuneability (I > Br > Cl ≫ F)^26,27^, allowing for the rational design of catalysts^28,29^, and supramolecular frameworks^30,31^. Halogen bonding is defined as the attraction between the region of elevated electrostatic potential^32^ on the halogen bond donor, associated with the electron-poor *σ*-hole^33^, and an electron-rich halogen bond acceptor, such as a Lewis base or nucleophile^34^. Recently, halogen bonding has been introduced in the field of supramolecular rotators, in configurations such as the molecular rotor on axle^35,36^ and a molecular top^37^. While halogen bonding served the role of an attractive interaction to maintain a particular geometry that best favors the rotation, the direct influence of halogen bonding contacts on the molecular dynamics was not the focus of these works.

Here, we show the first instance of halogen bonding having a direct influence on local dynamical processes, with the halogen bond donor acting as a dynamics catalyst superior to the hydrogen bond. In this context, dynamics catalysis refers to the reduction of the energy barrier associated with a dynamic process, such as rotation. To this end, we employ deuterium solid-state NMR spectroscopy and spin-lattice relaxation time constant (*T*_1_) measurements on a series of cocrystals and salts featuring the halogen bond acceptor 2,3,5,6-tetramethylpyrazine (TMP), observing changes in the methyl group activation energy upon the introduction of a halogen bond or hydrogen bond, and comparing the results to those obtained for pure TMP and its halide salts (HCl, HBr). The TMP acceptor provides an ideal scaffold to study the effect of changes in the chemical environment on rotation, as the methyl groups are consistently located immediately adjacent to the non-covalent interaction being introduced.

## Results

### Structural elements

**Fig. 1:**
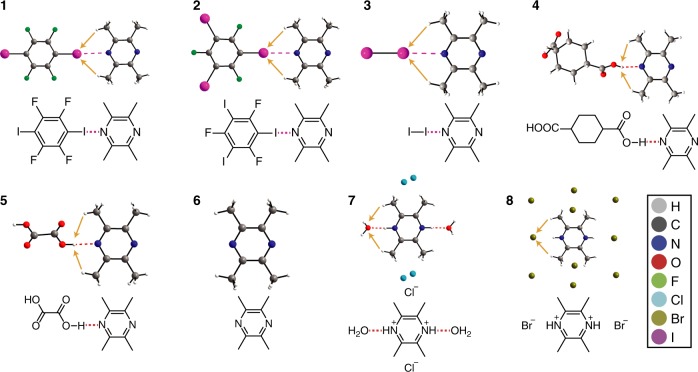
Depictions of the X-ray crystal structures and molecular structures involving TMP (**1**–**8**) surveyed in this work. The dashed magenta lines denote the halogen bonds while the dashed red lines denote hydrogen bonds. The orange arrows denote close contacts between the protons on the methyl group and nearby atoms

Samples **1**–**3**, shown in Fig. 1, consist of cocrystals featuring a I···N halogen bond [**1** (1,4-diiodotetrafluorobenzene)(TMP)^38^; **2** (1,3,5-trifluoro-2,4,6-triiodobenzene)(TMP)^39^; **3** (iodine)(TMP)]^40^, while samples **4** and **5** feature a O–H···N hydrogen bond [**4** (1,4-cyclohexanedicarboxylic acid)(TMP)^41^; **5** (oxalic acid)(TMP)^41^]. Sample **6** consists of pure TMP (**6**, TMP)^42^ and serves as a reference point for the activation energy, while the halide salts [**7** (2,3,5,6-tetramethylpyrazinium hydrochloride hydrate)^43^, **8** (2,3,5,6-tetramethylpyrazinium hydrobromide)^43^] serve as controls for the presence of non-halogen-bonded halogens near the methyl groups. Unfortunately, the sample quality of the hydroiodide salt^43^ was not satisfactory despite multiple preparatory attempts, and was therefore excluded from our analysis. The crystal structures mentioned here have been described elsewhere, with the halogen bond and hydrogen bond geometries summarized in Table 1. In brief, the halogen bond geometries are comparable across samples **1**, **2**, and **3**, with **2** having the shortest halogen bond and **3** having the longest halogen bond, placing an iodine atom in proximity to the rotating methyl groups of the acceptor. The hydrogen bonding geometries are also similar in samples **4** and **5**, although **4** has a hydrogen bond ~0.1 Å longer than that of sample **5**, with an O–H group in proximity to the methyl groups. Compound **7** is a hydrate, with the water molecule interacting with the N–H^+^ moiety via a N–H···O hydrogen bond. The Cl^−^ anions in compound **7** occupy the spaces near the center between both methyl groups. In contrast, compound **8** is not a hydrate, with the Br^−^ anions distributed around the unit cell. Additional Cl^−^ and Br^−^ anions are shown in Fig. 1 for compounds **7** and **8**, respectively, to show the environment surrounding the methyl groups of interest. Across each sample, contacts to the methyl groups vary, with no clear trend in terms of distance or number of contacts. As a result, this series of compounds presents an ideal case enabling the assessment of the impact of a halogen bonding or hydrogen bonding moiety on the methyl group rotations.

**Table 1:** Methyl group rotational activation energy for each compound derived from the *T*_1_ relaxation experiments

Compound	I···N/H···N bond length (Å)	Activation energy (kJ mol^−1^)	Note
1	3.0665(18)	2.84 ± 0.14	Halogen bonded
2	2.993(3)	3.24 ± 0.13	Halogen bonded
3	3.075(5)	3.62 ± 0.13	Halogen bonded
4	1.919	4.17 ± 0.09	Hydrogen bonded
5	1.809	5.02 ± 0.06	Hydrogen bonded
6	n/a	7.31 ± 0.17	Pure TMP
7	n/a	6.81 ± 0.16	HCl salt
8	n/a	13.64 ± 0.45	HBr salt

### Experimental activation energies

Solid-state NMR spectroscopy of deuterium, a quadrupolar nucleus (^2^H, spin *I* = 1), has long been used for characterizing molecular dynamics. Under static sample conditions, the ^2^H solid-state NMR spectral line shape for a methyl group is diagnostic of its motion, with rapid motions typically causing an averaging effect and narrowing the spectra. Further, the quadrupolar coupling and lower magnetogyric ratio of ^2^H renders it ideal for studies on dynamics, in contrast to ^1^H (spin *I* = ½) that have NMR spectra typically dominated by dipolar coupling in the solid state. With a natural abundance of 0.0115%, isotopic enrichment is used to perform ^2^H NMR experiments. This provides an opportunity to selectively label the methyl groups of interest here. Satisfactory enrichment of TMP was obtained through deuterium-proton exchange in a hot alkaline solution over one week, yielding a consistent deuterium enrichment factor across all samples.

**Fig. 2:**
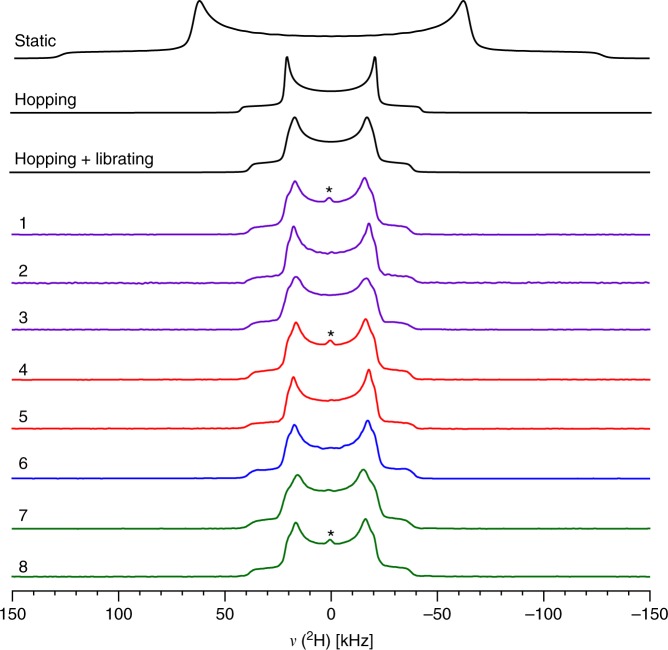
Experimental static deuterium (^2^H) solid-state NMR spectra of compounds **1**–**8** at room temperature. In black are the simulated spectra, in purple are the halogen bonded species, in red are the hydrogen bonded species, in blue is pure TMP, and the halide salts are in green. The asterisks denotes a trace quantity (<1%) of D_2_O

The ^2^H solid-state NMR spectra acquired at room temperature for each sample using a quadrupolar echo pulse sequence^44^ are shown in Fig. 2, with the linewidths given in Supplementary Table 2. These experiments were carried out on stationary powdered samples. The resulting spectra are superimposable, suggesting unsurprisingly that the global nature of the methyl group dynamics is unchanged across the various samples. In order to extract a full and detailed description of the dynamics, the ^2^H line shapes were simulated using EXPRESS^45^ software. The spectra were fit to a threefold hopping model along with additional librational motions of the CH_3_ groups. This libration, also qualitatively reproduced by DFT calculations (see Supplementary Figure 5), is rationalized by the rotation of the adjacent methyl group, causing an increase in the distance between both methyl groups in the gauche position as a result of steric effects. While the rapid rotation is responsible for narrowing the line shape, the libration is manifested as a broadening of the main spectral discontinuities (see the simulations in Fig. 2 and Supplementary Figures 3 and 4). Accordingly, the narrow line shape (35 ± 3 kHz horn separation) is consistent with rapidly rotating methyl groups, while there is an absence of other major dynamic processes such as a ring-flip of TMP along the halogen or hydrogen bond axis. In order to extract the thermodynamic parameters governing the methyl group rotation, the ^2^H spin-lattice (*T*_1_) relaxation time constants have been measured by solid-state NMR spectroscopy over a wide temperature range of ~140 °C at 20 °C intervals. These measurements were achieved by combining the quadrupolar echo^44^ pulse sequence with the inversion recovery experiment. The *T*_1_ relaxation time constants were measured using the integral of the entire line shape, and were determined to be nearly isotropic by measuring the *T*_1_ values for separate parts of the spectrum. Furthermore, throughout the temperature range, apart from sample **8**, the ^2^H line shape remained unchanged down to ~150 K, supporting an Arrhenius-dominated motion in the experimental temperature range^46^. A previous study on the *T*_1_ relaxation time constants of pure TMP reveals that quantum tunneling is insignificant above temperatures of 80 K^47^. Consequently, in the high temperature regime, the Arrhenius activation energy can be conveniently extracted using Eq. 1:^48^

1 $$T_1 \propto \frac{1}{{\tau _{\mathrm{c}}}} = A\exp \left( {\frac{{ - E_{\mathrm{a}}}}{{RT}}} \right)$$

**Fig. 3:**
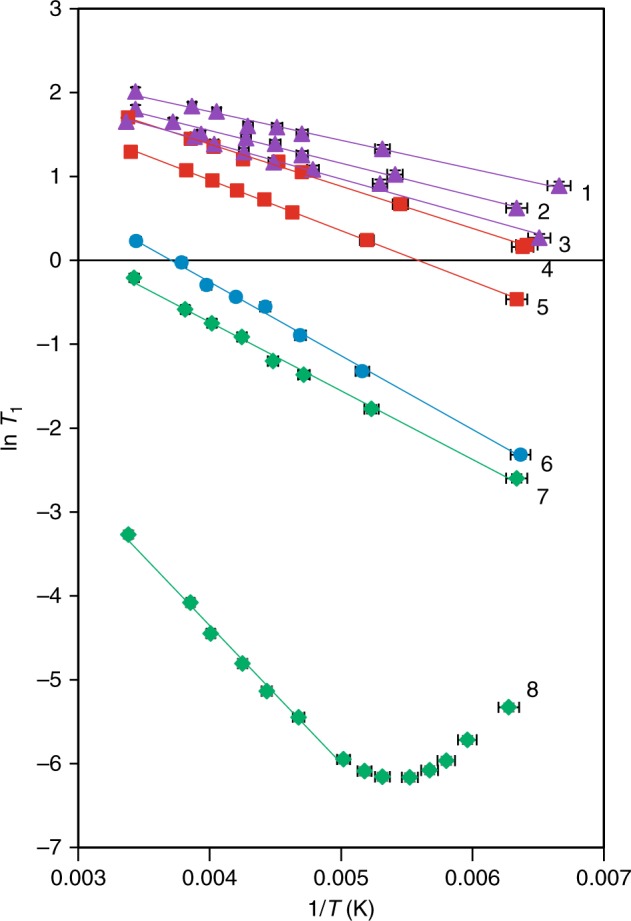
Experimental ln *T*_1_ relaxation times plotted as a function of the inverse temperature (1/*T*). The error bars represent an upper limit on instability in the temperature and the uncertainty of the *T*_1_ relaxation time measurements

In Eq. 1, *τ*_c_ corresponds to the correlation time, *A* is the pre-exponential factor, *E*_a_ is the Arrhenius activation energy, *R* is the gas constant, and *T* is the temperature. The activation energy can be extracted by plotting the natural logarithm of the *T*_1_ relaxation time constant as a function of the inverse temperature (1/*T*), with the slope equal to −*E*_a_/*R*. These data are plotted in Fig. 3 and tabulated in Supplementary Tables 3–10, with the fitting parameters given in Supplementary Table 1. Upon cooling sample **8** to 180 K, a line shape distortion was observed, which is associated with approaching the *T*_1_ minimum. Consequently, supplementary data point sampling was performed for sample **8** at these lower temperatures, and the activation energy was extracted using data at higher temperatures. The activation energies are summarized in Table 1.

The activation energies associated with the methyl group rotation are lower for the cocrystals featuring a halogen bond (**1**–**3**) and a hydrogen bond (**4** and **5**) when compared to pure TMP (**6**). Moreover, the activation energies for the halogen bonded complexes were found to be significantly lower than those of the hydrogen bonded complexes, with **1** having an activation energy of nearly half that of **5**, for example. Halide salt **7**, which has a water molecule in proximity of the methyl groups, has a slightly lower activation energy when compared to the pure acceptor, while **8** has the highest activation energy, at 186% more than that of pure TMP.

### Computational chemistry

To support the experimental results, a series of DFT calculations was carried out using the Amsterdam Density Functional (ADF)^49–51^ software package on the cocrystals featuring perfluorinated halogen bond donors, in order to provide a theoretical basis for the observed reductions in the activation energy. The models of **1** and **2** consisted of TMP interacting with one nearby halogen bond donor, identical to the crystal structure depictions shown in Fig. 1. A series of linear transit calculations have been performed, altering the H–C–C–C dihedral angle from −180° to 90°, in steps of 2.5°, for the methyl group closest to the halogen bond donor. The energy was calculated at every point of the methyl group rotation after a geometry optimization cycle, while maintaining a constrained dihedral angle. The calculated activation energies were approximated by subtracting the energy minimum from the energy maximum. For both models, the energy minimum of a single methyl group rotation consisted of the staggered conformation (methyl groups 180° out of phase), while the energy maximum consisted of the gauche conformation (methyl groups in-phase). As a result of only rotating a single methyl group, the energy associated with that rotation could be estimated.

**Fig. 4:**
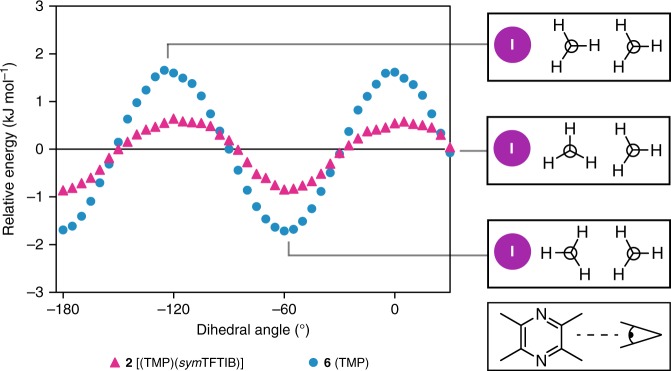
DFT-calculated relative energy as a function of the methyl group dihedral angle (H–C–C–C). The blue circles denote pure TMP (**6**) and the magenta triangles denote cocrystal **2**. A diagram showing the methyl group geometry at the energy maximum, zero-crossing, and minimum is shown on the right. A depiction showing the perspective in the diagram is shown on the bottom right

The results of the DFT calculations are shown in Fig. 4, comparing the relative energy of compounds **2** and **6**, upon the rotation of the methyl group. The results comparing structure **1** to structure **6** are shown in Supplementary Figure 6. The pseudo-sinusoidal nature of the energy upon the rotation of the methyl group is consistent with a *C*_3_ rotation, with three energy minima in a full 360° rotation due to the presence of three hydrogen atoms on the methyl group. Upon the introduction of a halogen bond, there is a clear increase in the energy minimum and a decrease in the energy maximum, with an overall energy barrier reduction of 58% for **1** and 56% for **2** when compared to pure TMP, in agreement with the experimental values. The orientation of the adjacent methyl group not interacting with iodine did not significantly impact the overall catalytic effect, with the results of the calculations shown in Supplementary Figure 7.

**Fig. 5:**
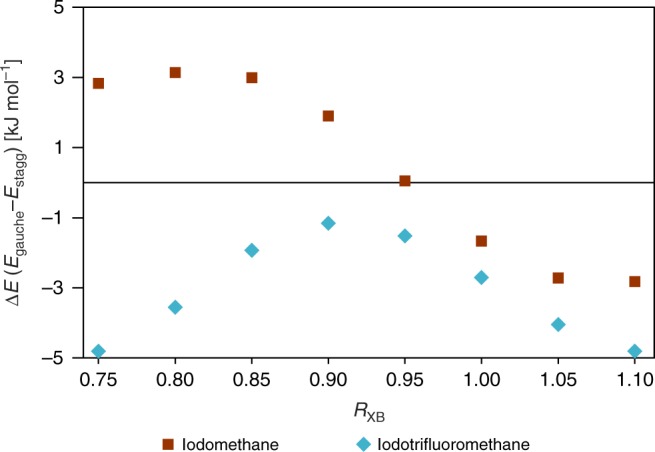
Calculated energy difference between the staggered and gauche conformations of TMP as a function of the I···N halogen bond length (expressed as *R*_XB_), interacting with either iodomethane (red squares) or iodotrifluoromethane (blue diamonds)

A series of calculations was then carried out comparing the effect of the halogen bond length (*d*_I···N_) on the methyl group activation energy, shown in Fig. 5. This was performed by calculating the energy difference between the in-phase and out-of-phase conformations on a model of TMP interacting with either iodomethane or iodotrifluoromethane, varying the distance between both fragments from an *R*_XB_ value of 1.10 to 0.75, at 0.05 increments. For clarity, here we use *R*_XB_ to refer to the quotient of the halogen bond length (*d*_I···N_) and the sum of their van der Waals radii (*R*_XB_ = *d*_I···N_/∑*d*_vdw_).

As shown in Fig. 5, the I···N distance plays a clear role in reducing the rotational activation energy barrier, with the catalytic abilities of the halogen bond being most pronounced between *R*_XB_ of 0.85 and 0.95. The difference in the energy curves between iodomethane and iodotrifluoromethane presented in Fig. 5 is quite substantial, suggesting that the catalytic effect associated with the halogen bond isn’t merely due to the presence of an iodine atom, but the choice of the donor moiety may also play a role. In the case of the non-fluorinated iodomethane, the energy barrier crosses the zero energy point at *R*_XB_ = 0.95, suggesting that the gauche conformation becomes energetically favorable rather than the staggered conformation at short I···N distance. In contrast, when interacting with iodotrifluoromethane, the staggered conformation remains energetically favorable as the halogen bond distance is decreased, reaching an energy minimum at an *R*_XB_ of 0.90. We refer to this effect as catalytic because the halogen bond is not consumed or destroyed during the process of methyl rotation.

## Discussion

**Fig. 6:**
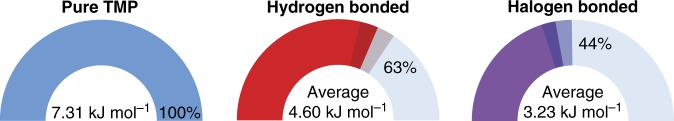
Summary of the reduction in the experimentally measured activation energy associated with the rotation of the methyl groups. The average activation energies are given for the halogen bonded and hydrogen bonded cocrystals

The experimental activation energies, summarized in Table 1 and Fig. 6, are lowest for the halogen bonded cocrystals (**1**–**3**), followed by the hydrogen bonded cocrystals (**4**, **5**), the pure TMP (**6**) and the halide salts (**7**, **8**). Furthermore, the activation energies of **1**–**3** are amongst the lowest in the literature for a methyl group rotation, comparable to 2,6-dimethylnaphthalene and 1,3,5-trichloro-2,4,6-trimethylbenzene, having activation energy of 1.6^18^ and 2.4 kJ mol^−1 ^^52^, respectively. In the case of the latter, the low energy barrier has been attributed to the chlorine substitution, in contrast to the 8.0 kJ mol^−1^ barrier observed in hexamethylbenzene^53^. As a point of reference, the rotational activation energy of a methyl group in organic molecules typically spans from 6.3 to 24 kJ mol^−1^ ^47,54^. The reduction in the methyl group activation energy observed in the cocrystals studied here is attributed to the presence of the halogen bond donor and hydrogen bond donor in proximity to the methyl groups, as no clear trends can be discerned relating the number of contacts to the methyl groups (see Supplementary Figures 1 & 2) or to the crystal packing. Furthermore, the mere presence of halogen atoms in proximity to the methyl groups was not sufficient to catalyze the methyl group rotation, with compound **8** instead exhibiting a significant increase in the activation energy. The slight decrease in the activation energy observed in compound **7** can be rationalized by the presence of a water molecule participating in a O···N contact near the methyl groups, despite the presence of chloride anions. As a result, the catalytic effect observed in compounds **1**–**3** appears to be unique to covalently bonded halogens.

The DFT results on isolated models of **1**, **2**, and **6** (Fig. 4 and Supplementary Figure 6) suggest that this catalytic effect associated with the halogen bond is a result of the destabilization of the staggered conformation and the stabilization of the gauche conformation. Consequently, the difference between the energy maximum and minimum is reduced in the halogen bonded cocrystals, causing a net reduction in the activation energy. This is in contrast to the previous theoretical investigations on the catalytic effect of van der Waals contacts involving hydrogen on methyl groups, revealing primarily an increase in the energy minimum^15^. A potential mechanism for the destabilization of the staggered conformation may be due to the repulsion between the protons of the methyl group, which carry a positive electrostatic potential (+74.5 kJ mol^−1^; see Supplementary Figure 9), and the positive *σ*-hole of the halogen bond donor. As the electrostatic potential between the protons of the methyl group is slightly negative (–5.7 kJ mol^−1^), this may cause an attractive interaction to the *σ*-hole of the halogen bond, leading to a stabilizing effect. In this respect, a larger *σ*-hole would lead towards a stronger hydrogen···iodine interaction, further reducing the rotational energy barrier. (Another less plausible mechanism for the reduction of the energy barrier could be related to the skeletal flexing of TMP upon halogen bonding. However, an inspection of the crystal structures shows that the structural changes induced upon halogen bonding are tiny (e.g., C–C–CH_3_ angle in TMP changes by 1° and distance between the two methyl carbons changes by ~0.01 Å). It is not clear how these changes would result in a lower activation energy in the halogen bonded systems.)

The DFT results suggest that the distance between the iodine and the methyl rotors plays an important role in determining the catalytic ability of the halogen bond, being most significant for *R*_XB_ values between 0.85 and 0.95. With an average *R*_XB_ value for the C–I···N fragment of 0.85 according to the Cambridge Structural Database^55^, these halogen bonds seem to be in the ideal range to act as a dynamical catalyst. When comparing the catalytic ability of iodomethane and iodotrifluoromethane as a function of the I···N distance by DFT calculations, a striking difference between both curves is observed, suggesting that the addition of electron withdrawing groups may alter the catalytic abilities of the halogen atom, and may allow for a tuneable dynamics catalyst. This is supported experimentally, with the catalytic ability of the halogen bond donor following the calculated molecular electrostatic potential of the *σ*-hole (see Supplementary Figure 8). The calculated potential of the *σ*-hole, highest in **1** at 168 kJ mol^−1^, followed by **2** at 162 kJ mol^−1^, and **3** at 155 kJ mol^−1^, satisfyingly follows the order of reduction in the methyl group rotational energy barrier.

In conclusion, we have shown how halogen bonding plays a direct role as a supramolecular dynamics catalyst through the use of ^2^H solid-state NMR relaxation experiments. In the cases shown here, the catalytic impact of the halogen bond surpasses that of the hydrogen bond. Computational support suggests that this is achieved by destabilizing the staggered conformation and stabilizing the gauche conformation, resulting in an overall reduction of the activation energy barrier. Further, this catalytic effect may be tuneable by changing the electron withdrawing substitutions adjacent to the halogen bond donor. Consequently, the halogen bond may not only be a versatile interaction useful in crystal engineering, but may bring a unique ability of promoting dynamics within functional molecules, such as enzymes, catalysts, and molecular machines.

## Methods

### Sample preparation

2,3,5,6-tetramethylpyrazine (98%), 1,4-diiodotetrafluorobenzene (99%), iodine (99%), oxalic acid (99%), and hydrobromic acid (48%) were purchased from Sigma Aldrich. 1,3,5-trifluoro-2,4,6-triiodobenzene was purchased from Alfa Aesar. 1,4-cyclohexanedicarboxylic acid (99%) was purchased from Acros Organics. Deuterium oxide (> 99% deuterium) was purchased from Cambridge Isotope Laboratories. Hydrochloric acid (~38%) and all solvents were purchased from Fisher Scientific.

2,3,5,6-tetramethylpyrazine was deuterated following a modified literature procedure^56^. To a 50 mL glass pressure tube, 35 mL of a 0.6 M NaOH solution in D_2_O was added, followed by the addition of 2.05 g of 2,3,5,6-tetramethylpyrazine. The glass vessel was placed in the oven at 90 °C, at which point the 2,3,5,6-tetramethylpyrazine dissolved completely, and left for 7 days. The glass pressure tube was removed from the oven and allowed to return to room temperature, allowing for the deuterated 2,3,5,6-tetramethylpyrazine to crystallize. The solution was filtered and rinsed with water. The product was sublimed (yield = 57%), affording 2,3,5,6-tetramethylpyrazine with a deuterium enrichment factor of ~60.1%. Compounds **1**^38^, **2**^39^, **7**^43^, and **8**^43^ were reproduced using their literature procedure using the deuterated product. Compound **3**^40^ was prepared by cosublimation^57^ by combining I_2_ and the deuterated 2,3,5,6-tetramethylpyrazine in a round bottom sublimation apparatus. Compounds **4**^41^ and **5**^41^ were prepared mechanochemically using a Retsch MM400 ball mill, by placing both the deuterated 2,3,5,6-tetramethylpyrazine and hydrogen bond donor at equimolar quantities in a 10 mL stainless steel ball mill jar. The products were ground for 30 min at a frequency of 25 Hz, using two stainless steel milling balls. The crystal structure for each powdered sample was verified by powder X-ray diffraction on a Rigaku Ultima IV instrument unless otherwise stated, with 2*θ* ranging from 5 to 65° in increments of 0.02° using Cu Kα radiation. The powder X-ray diffractograms are shown in Supplementary Figures 10 to 17.

### ^2^H solid-state NMR

All ^2^H solid-state NMR experiments were performed at 4.7T (ν_L_(^2^H) = 30.7 MHz) using a Bruker Avance III console and a single-channel wideline probe fitted with a 5 mm coil. Static ^2^H spectra were acquired using a quadrupolar echo^44^ (π/2–τ–π/2–τ) pulse sequence, using a 4.7 μs π/2 pulse length, and a 20 μs τ delay. The *T*_1_ relaxation experiments were performed using a quadrupolar echo coupled with an inversion recovery sequence at variable temperatures. The temperature was controlled by flowing dry nitrogen gas through a heat exchanger submerged in liquid nitrogen, and subsequently heated inside the probe to the desired temperature. The temperature was regulated using a Bruker variable temperature controller with the thermocouple placed near the sample, while the sample temperature was measured by placing a copper/constantan thermocouple directly on the RF coil, while using an ice water bath as a reference.

### Computational

The DFT calculations were performed using the Amsterdam Density Functional software (ADF 2017)^49–51^. Models were built from the experimental atomic coordinates taken from the crystal structures, featuring one halogen bond donor interacting with 2,3,5,6-tetramethylpyrazine. Each model was initially optimized using the TZ2P basis set, accounting for dispersion forces using Grimme3 BJDAMP^58^ and relativistic effects using ZORA^59^. Linear transit calculations were performed using the TZ2P basis set by rotating the methyl group at steps of 2.5° relative to the H–C–C–C dihedral angle, performing a geometry optimization routine at each step, and calculating the energy. Single point energy calculations were performed using pre-optimized models of the staggered and gauche conformers with variable distances and halogen bond donor fragments. The QZ4P basis set was used for all iodine atoms in the linear transit and single point energy calculations. The molecular electrostatic surface potential surfaces were generated using Gaussian 09^60^ with the B3LYP functional and the 3-21G basis set, and visualized using GaussView^61^ at 0.002 e a.u.^−1^. The resulting molecular electrostatic potential values for the *σ*-hole were found to be in agreement with previous reports^62^.

## Supplementary information

Supplementary Information

## Data Availability

The authors declare that all data supporting the findings of this study are available within the paper and its supplementary information files.
