# Supplementary material for: Halogen bonding as a supramolecular dynamics catalyst
Source: Nat Commun. 2019 Feb 22;10:916. doi: 10.1038/s41467-019-08878-8 (PMC6385366; doi:10.1038/s41467-019-08878-8)
Supplement: Supplementary file 1 — Supplementary Information [file 41467_2019_8878_MOESM1_ESM.pdf]

Supplementary Information

Halogen Bonding as a Supramolecular Dynamics Catalyst

Patrick M.J. Szell, Scott Zablotny, and David L. Bryce*

*Author to whom correspondence is to be addressed

Department of Chemistry and Biomolecular Sciences & Centre for Catalysis Research and Innovation

University of Ottawa

10 Marie Curie Private

Ottawa, Ontario K1N 6N5

Canada

Tel: +1-613-562-5800 ext.2018; fax: +1-613-562-5170

Email: dbryce@uottawa.ca

1.0 – Methyl Group Contact Analysis

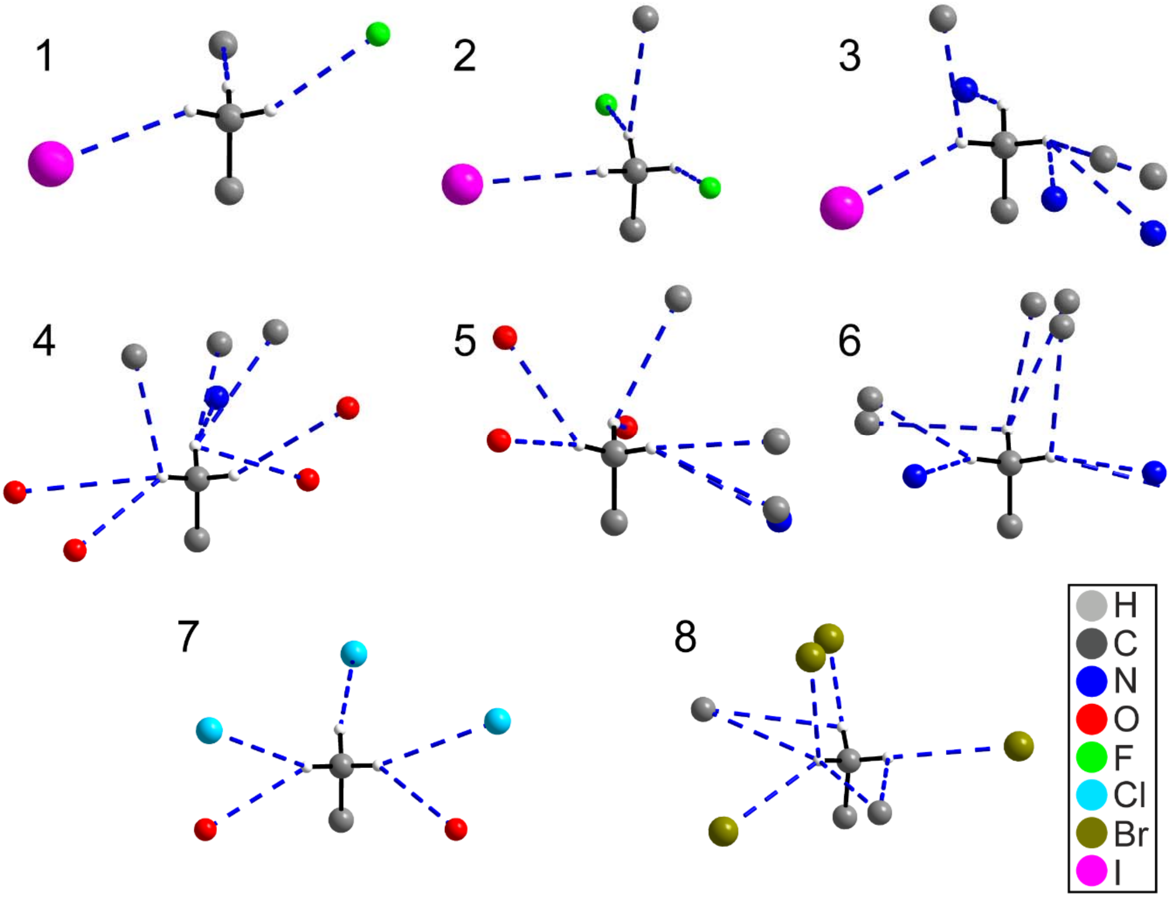

Supplementary Figure 1. Depiction showing the intermolecular contacts within 3.3 Å of the protons of a methyl group in their respective crystal structure. Hydrogen···hydrogen contacts are not shown for clarity. The same structures with the inclusion of hydrogen···hydrogen contacts can be seen in Supplementary Figure 1.

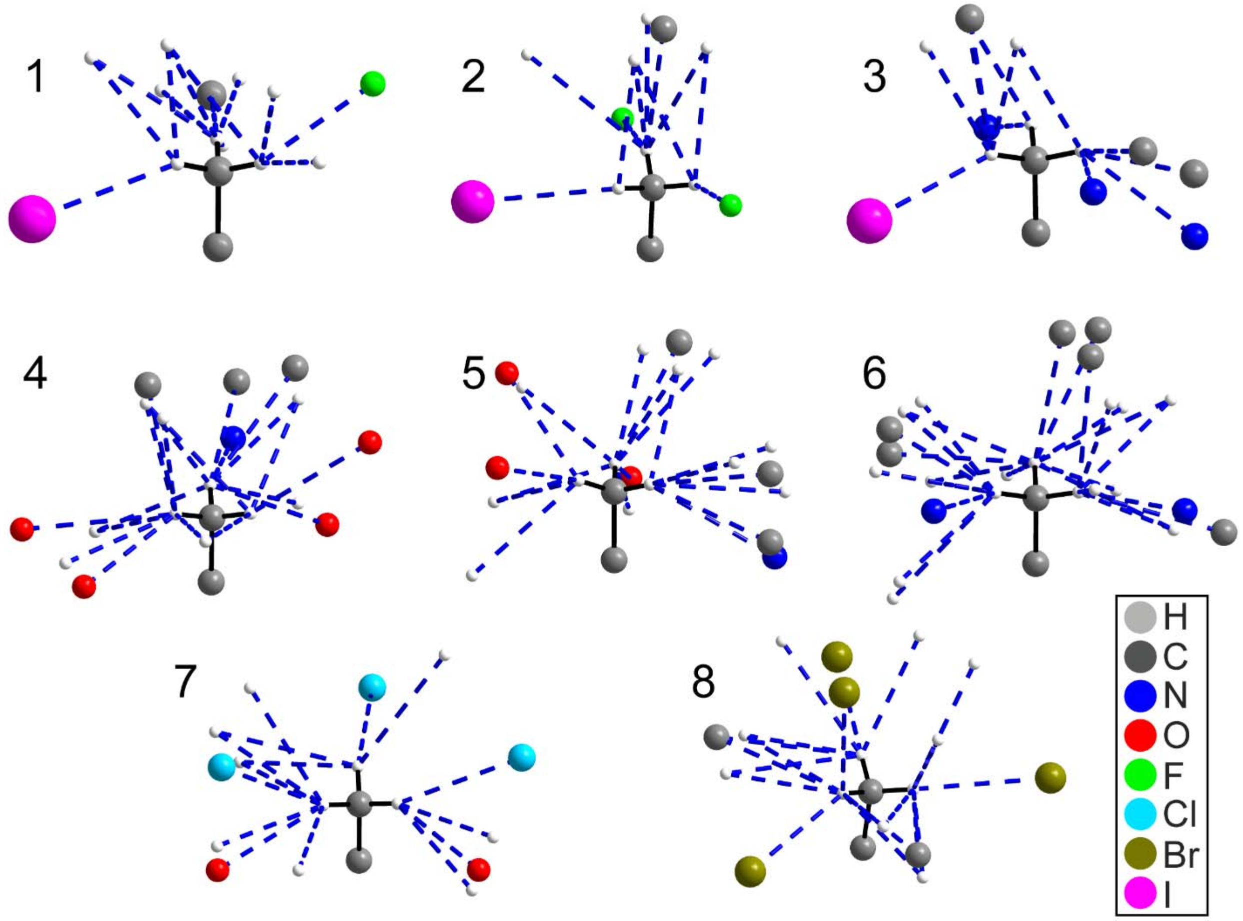

Supplementary Figure 2. Depiction showing the intermolecular contacts within 3.3 Å of the protons of a methyl group in their respective crystal structure. The above figure is identical to Supplementary Figure 1, but includes hydrogen···hydrogen contacts.

2.0 – Supporting EXPRESS Simulations

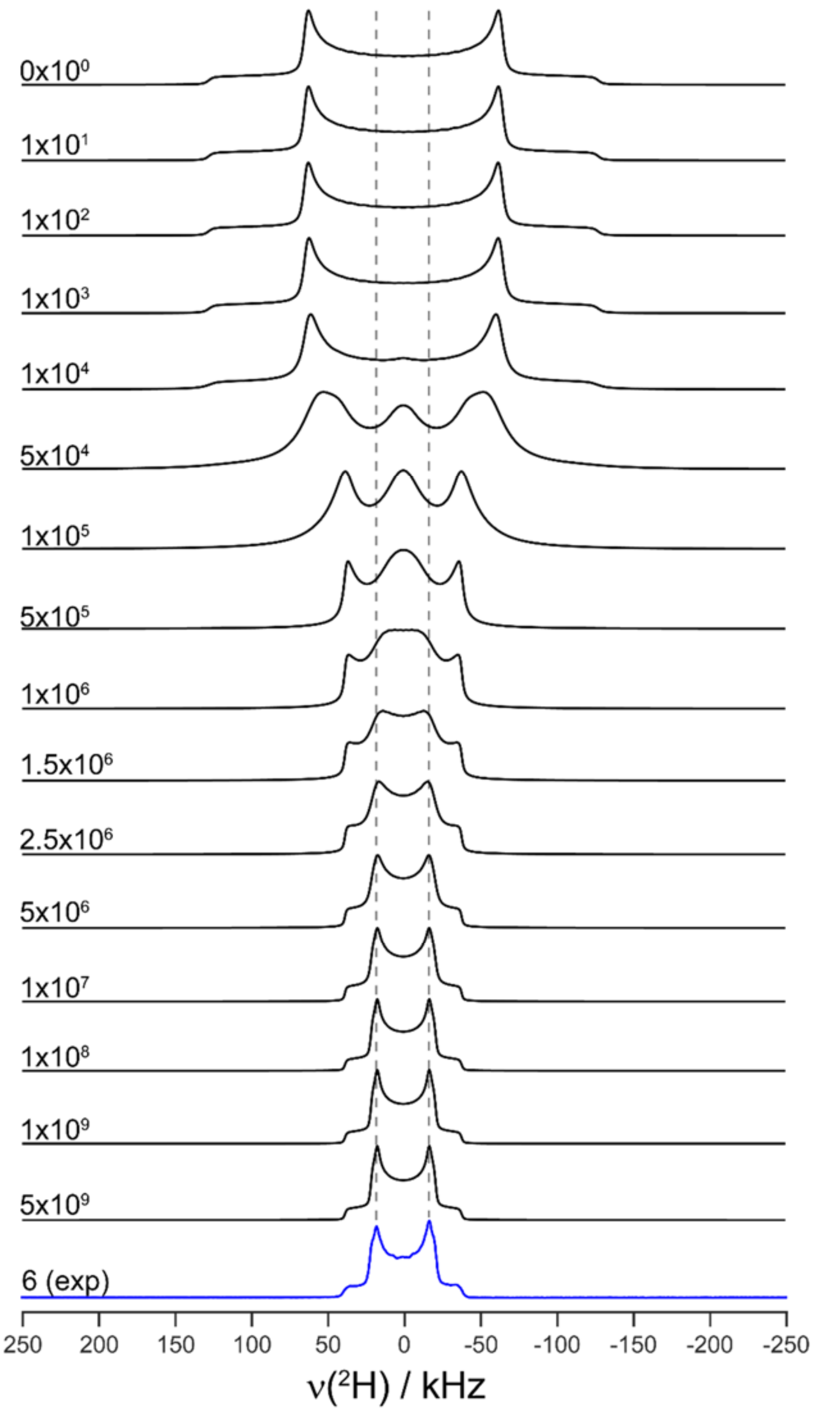

Supplementary Figure 3. Hydrogen-2 line shape simulations under static conditions for a methyl group exhibiting both rotation and libration, varying the rate constant (denoted on the left) for both movements from 0 to $5x10^9$. The simulations were performed using EXPRESS.[1] The experimental ^2H spectrum for sample **6** is shown in blue. The dashed line serves as a guide.

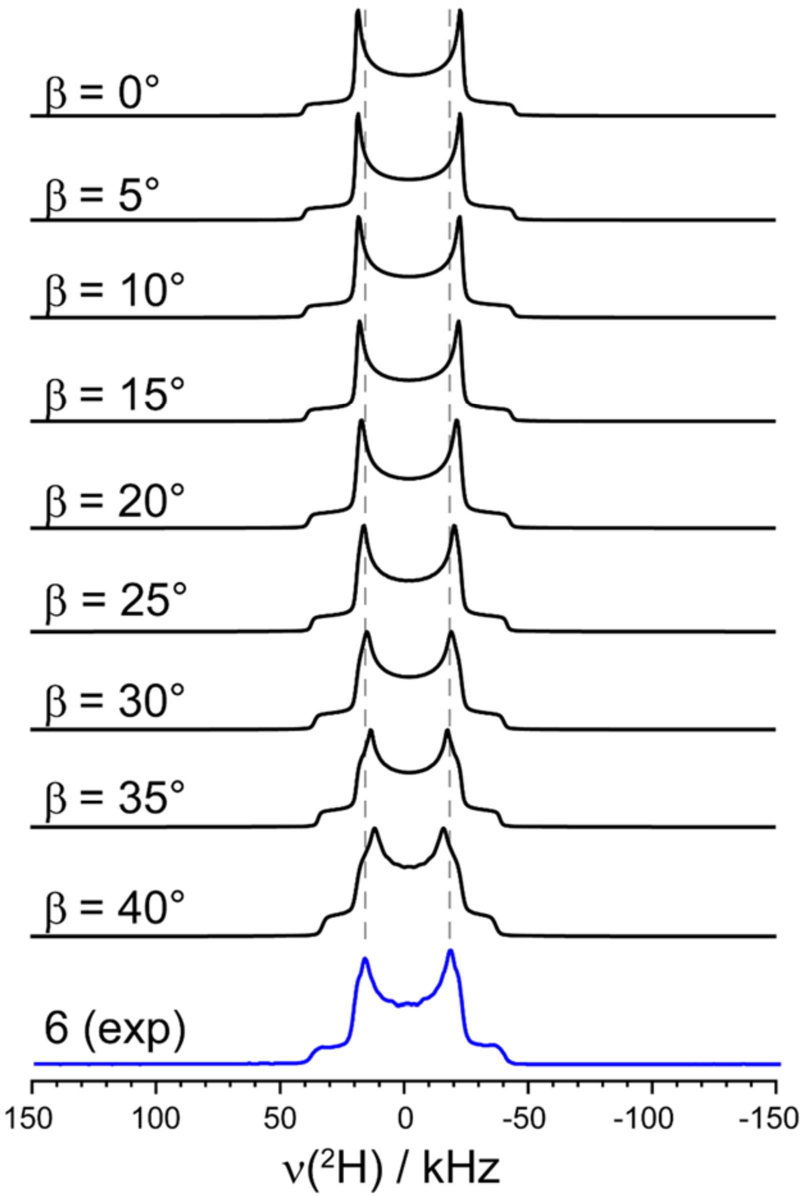

Supplementary Figure 4. Hydrogen-2 line shape simulations under static conditions for a methyl group exhibiting both rotation and libration, varying the methyl group libration angle from β = 0° to β = 40°. The simulations were performed using EXPRESS.[1] The experimental ^{2}H spectrum for sample **6** is shown in blue. The dashed line serves as a guide.

Supplementary Methods: Express simulation parameters. Express simulations were performed using experimental conditions (ν_L, pulse length, delays), a 3-fold hopping model [(α_1, β_1, γ_1) = (0, 70.5, 0); (α_2, β_2, γ_2) = (0, 70.5, 120); (α_3, β_3, γ_3) = (0, 70.5, 240)] with simultaneous libration [(α_1, β_1, γ_1) = (0, 0, 0); (α_2, β_2, γ_2) = (0, 30, 0)] at a rate of $k = 5 \times 10^9$, with C_Q =170 kHz and $\eta = 0$. The simulations in Supplementary Figure 3 were obtained by varying k, while the simulations of Supplementary Figure 4 were obtained by varying β_2 of the methyl group libration.

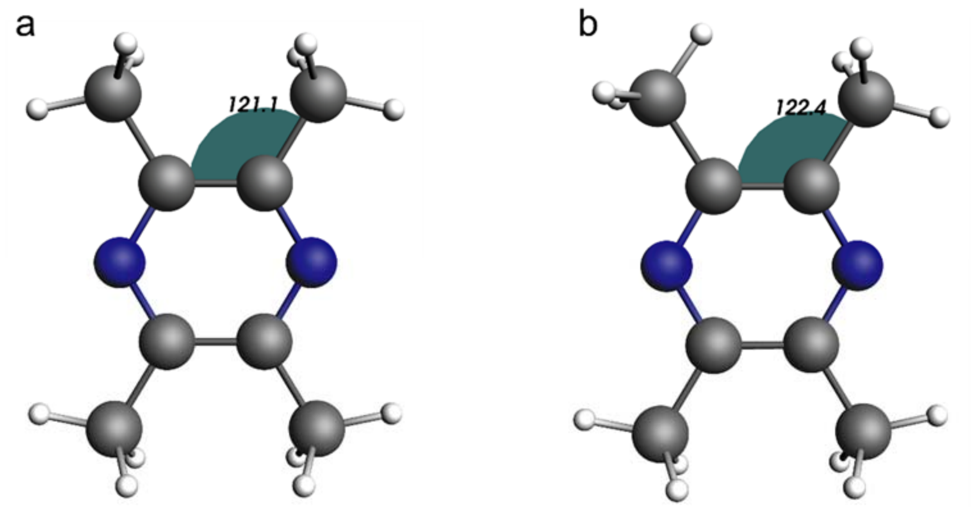

Supplementary Figure 5. ADF simulation[2] of sample **6** upon the rotation of a single methyl group, showing the libration of the adjacent methyl group as a result of the methyl rotation. The staggered conformation is shown in **a**, and the gauche conformation is shown in **b**.

3.0 - Variable Temperature T_1 Relaxation Time Constant Measurements.

Supplementary Table 1 Variable temperature T_1 relaxation time constant fitting parameters for compound **1** to **8**.

compound	activation energy (kJ mol^{-1})	ln(A)[a]	R^2 [b]	note
1	2.84 ± 0.14	3.14 ± 0.08	0.989	halogen bonded
2	3.24 ± 0.13	3.11 ± 0.07	0.991	halogen bonded
3	3.62 ± 0.13	3.15 ± 0.07	0.993	halogen bonded
4	4.17 ± 0.09	3.39 ± 0.05	0.997	hydrogen bonded
5	5.02 ± 0.06	3.37 ± 0.03	0.999	hydrogen bonded
6	7.31 ± 0.17	3.26 ± 0.09	0.997	pure TMP
7	6.81 ± 0.16	2.54 ± 0.09	0.997	HCl salt
8	13.64 ± 0.45 [c]	2.21 ± 0.23 [c]	0.995 [c]	HBr salt

[a] Arrhenius pre-exponential factor from eq. 1.
[b] Coefficient of determination.
[c] Fit using the T_1 relaxation time constants measured between 199 K and 295 K.

Supplementary Table 2 Observed separation (in kHz) between the horns of the Pake doublet for each spectrum at room temperature.

compound	Δν / kHz[a]	note
1	33 ± 1	halogen bonded
2	35 ± 1	halogen bonded
3	33 ± 1	halogen bonded
4	33 ± 1	hydrogen bonded
5	35 ± 1	hydrogen bonded
6	35 ± 1	pure TMP
7	32 ± 1	HCl salt
8	33 ± 1	HBr salt

Supplementary Table 3 T_1 relaxation time constant at various temperature increments for compound **1**.

Temperature (K) (± 2 K)	T_1 Relaxation time constant (s) (± 5%)
150.2	2.437
188.1	3.779
212.6	4.517
221.6	4.882
233.0	4.963
246.8	5.891
258.7	6.297
291.1	7.488

Supplementary Table 4 T_1 relaxation time constant at various temperature increments for compound **2**.

Temperature (K) (± 2 K)	T_1 Relaxation time constant (s) (± 5%)
157.8	1.869
184.8	2.785
212.6	3.510
222.2	4.004
233.8	4.292
254.1	4.502
268.8	5.204
291.1	6.079

Supplementary Table 5 T_1 relaxation time constant at various temperature increments for compound **3**.

Temperature (K) (± 2 K)	T_1 Relaxation time constant (s) (± 5%)
153.6	1.308
188.8	2.496
208.9	2.952
222.8	3.220
234.7	3.629
247.9	3.995
257.4	4.360
297.2	5.223

Supplementary Table 6 T_1 relaxation time constant at various temperature increments for compound **4**.

Temperature (K) (± 2 K)	T_1 Relaxation time constant (s) (± 5%)
155.9	1.203
156.7	1.173
183.5	1.954
212.6	2.865
221.1	3.242
235.0	3.334
248.2	3.865
259.3	4.265
296.0	5.505

Supplementary Table 7 T_1 relaxation time constant at various temperature increments for compound **5**.

Temperature (K) (± 2 K)	T_1 Relaxation time constant (s) (± 5%)
157.8	0.629
192.4	1.272
216.0	1.770
226.4	2.070
237.6	2.301
248.7	2.597
261.6	2.925
294.2	3.643

Supplementary Table 8 T_1 relaxation time constant at various temperature increments for compound **6**.

Temperature (K) (± 2 K)	T_1 Relaxation time constant (s) (± 5%)
157.1	0.099
193.7	0.267
213.2	0.410
226.1	0.575
238.1	0.646
251.4	0.744
264.0	0.976
290.6	1.261

Supplementary Table 9 T_1 relaxation time constant at various temperature increments for compound **7**.

Temperature (K) (± 2)	T_1 Relaxation time constant (s) (± 5%)
157.8	0.074
191.1	0.169
212.0	0.256
223.1	0.301
235.6	0.401
249.0	0.473
262.2	0.557
292.1	0.813

Supplementary Table 10 T_1 relaxation time constant at various temperature increments for compound **8**.

Temperature (K) (± 2)	T_1 Relaxation time constant (s) (± 5%)
159.3	0.0049
167.8	0.0033
172.4	0.0026
176.2	0.0023
181.1	0.0021
188.1	0.0021
193.1	0.0023
199.2	0.0026
213.8	0.0043
225.5	0.0059
235.3	0.0082
249.5	0.0117
259.5	0.0169
295.7	0.0381

3.0 – Computational Support

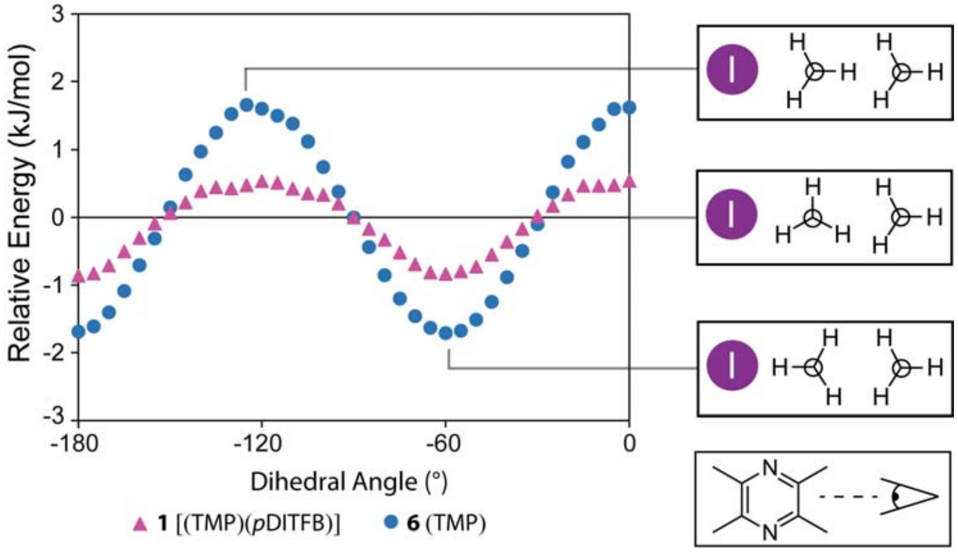

Supplementary Figure 6. DFT-calculated relative energy as a function of the methyl group dihedral angle (H-C-C-C). The blue circles denote pure TMP (**6**), whereas the magenta triangles denote the cocrystals featuring *p*DITFB (**1**). A diagram showing the orientation of both methyl groups at the energy maximum, zero-point, and minimum is shown on the right. A depiction showing the perspective in the diagram is shown on the bottom right. The rotational energy barrier of **6** is 3.34 kJ mol^{-1}, whereas the rotation energy barrier of **1** is of 1.39 kJ mol^{-1} for an overall energy reduction of 58%.

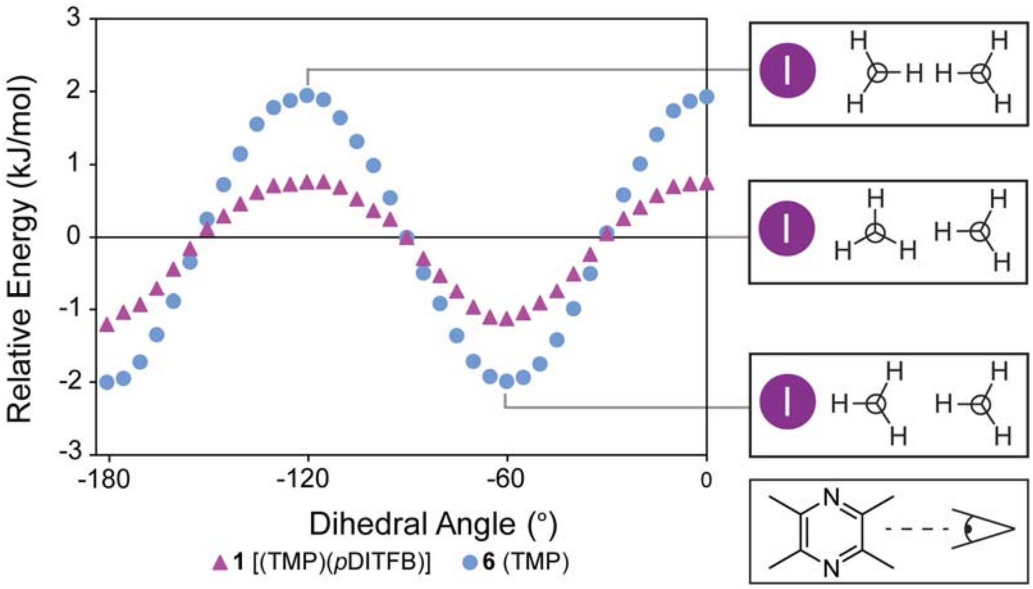

Supplementary Figure 7. DFT-calculated relative energy as a function of the methyl group dihedral angle (H-C-C-C). The blue circles denote pure TMP (**6**), whereas the magenta triangles denote the cocrystals featuring *p*DITFB (**1**). A diagram showing the orientation of both methyl groups at the energy maximum, zero-point, and minimum is shown on the right. A depiction showing the perspective in the diagram is shown on the bottom right. The calculations shown in Supplementary Figure 7 are the same as in Supplementary Figure 6 but with the second methyl group reoriented. The rotational energy barrier of **6** in this conformation is 3.95 kJ mol^{-1}, whereas the rotation energy barrier of **1** in this conformation is of 1.97 kJ mol^{-1} for an overall energy reduction factor of 50%.

3.1 – Calculated Molecular Electrostatic Potential Surface

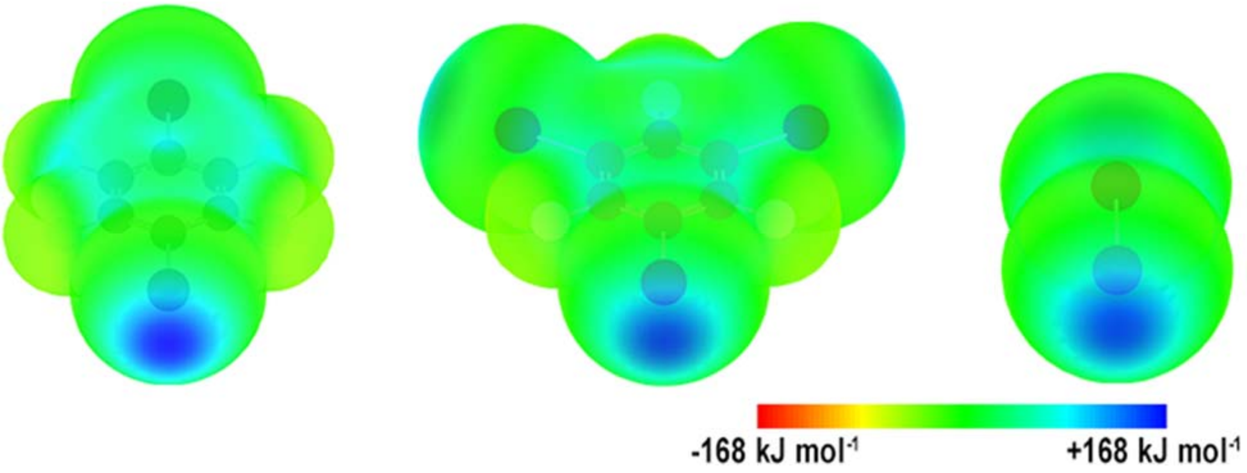

Supplementary Figure 8. Calculated molecular electrostatic potential surface generated at 0.002 e a.u.$^{-1}$ of 1,4-diiodotetrafluorobenzene (*p*DITFB, 168 kJ mol^{-1}), 1,3,5-trifluoro-2,4,6-triiodobenzene (*sym*TITFB, 162 kJ mol^{-1}), and iodine (I_2, 155 kJ mol^{-1}). The positive region in blue corresponds to the σ-hole.

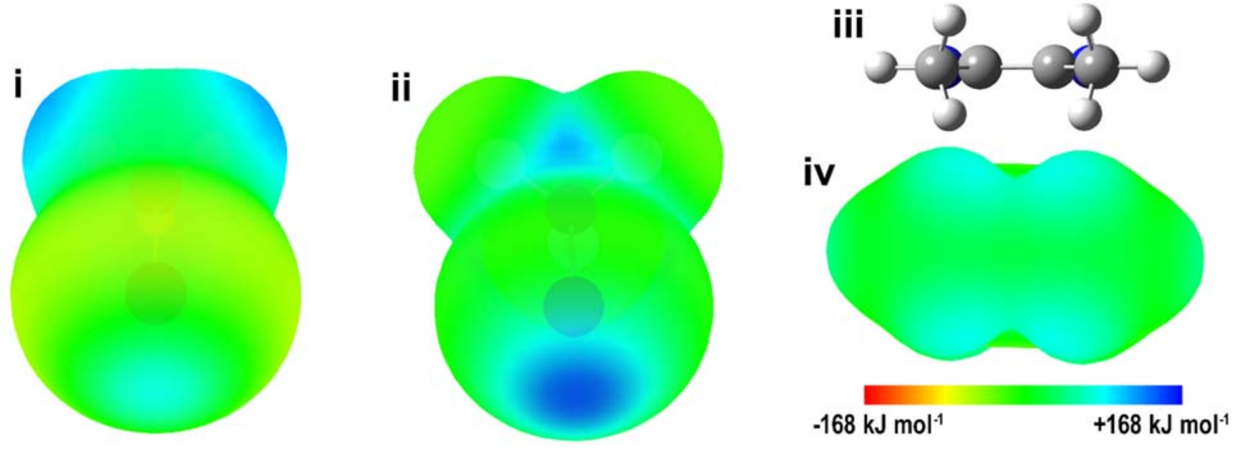

Supplementary Figure 9. Calculated molecular electrostatic potential surface generated at 0.002 e a.u.$^{-1}$ of (i) iodomethane (76.6 kJ mol^{-1}), (ii) trifluoroiodomethane (right, 149.7 kJ mol^{-1}), and (iv) 2,3,5,6-tetramethylpyrazine. The diagram in (iii) shows the orientation of the 2,3,5,6-tetramethylpyrazine molecule in iv. The positive region of the iodine atom in blue corresponds to the σ-hole.

4.0 - Powder X-ray Diffraction.

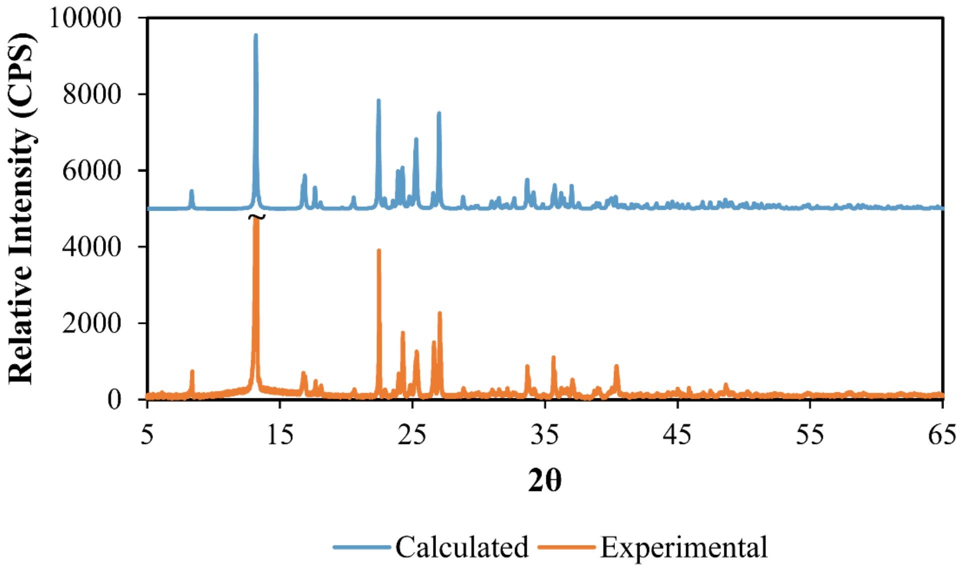

Supplementary Figure 10. Experimental and calculated powder X-ray diffractogram of compound **1** [(2,3,5,6-tetramethylpyrazine)(1,4-diiodotetrafluorobenzene)]. The powder X-ray was acquired on a PANalytical Empyrean using the same acquisition parameters. Baseline correction was implemented. The tilde over the diffraction at 13° indicates that it was cut for proper scaling.

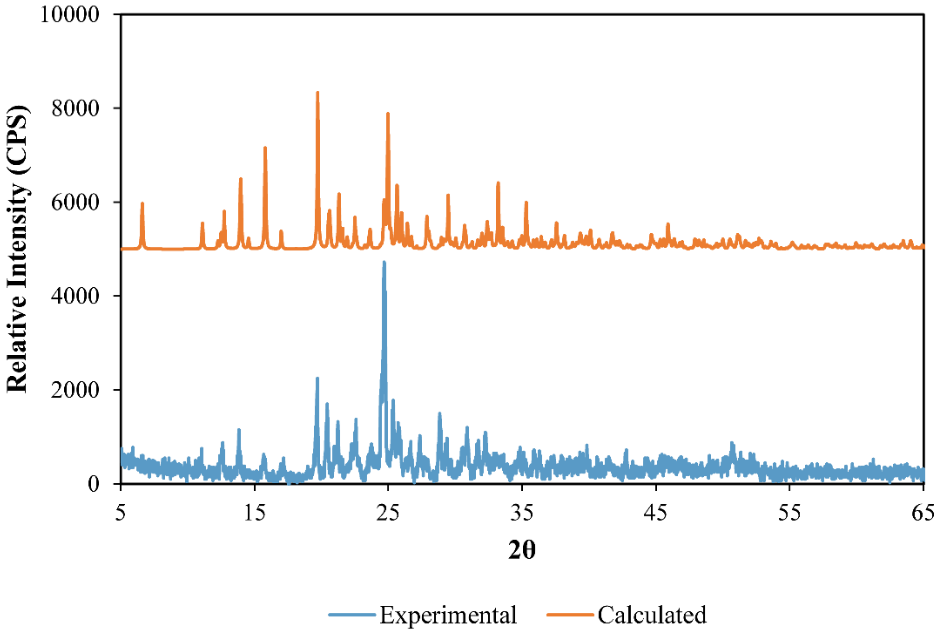

Supplementary Figure 11. Experimental and calculated powder X-ray diffractogram of compound **2** [(2,3,5,6-tetramethylpyrazine)(1,3,5-trifluoro-2,4,6-triiodobenzene)].

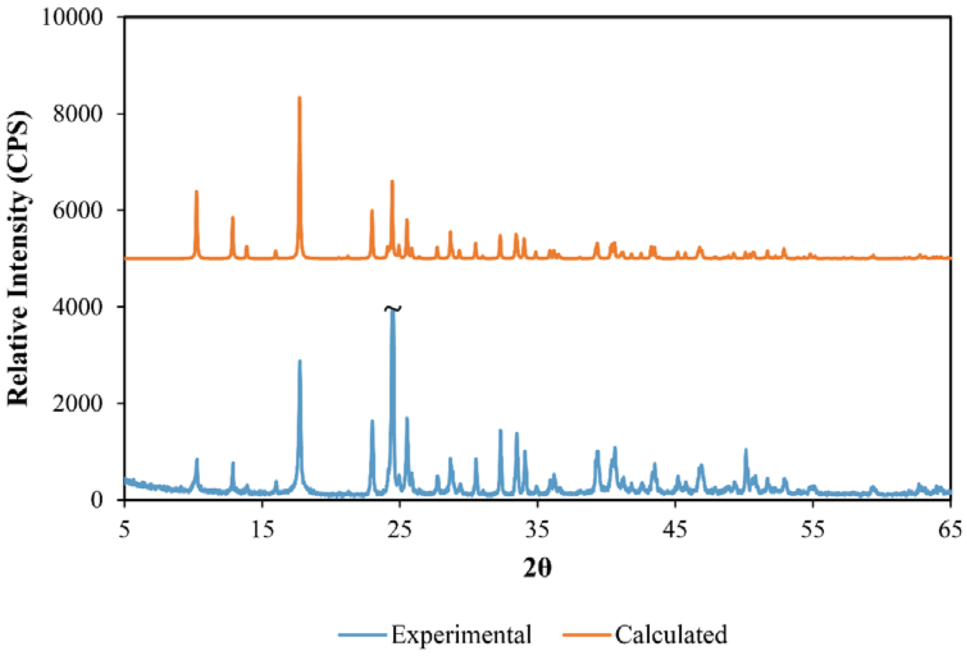

Supplementary Figure 12. Experimental and calculated powder X-ray diffractogram of compound **3** [(2,3,5,6-tetramethylpyrazine)(iodine)]. The tilde over the diffraction at 24º indicates that it was cut for proper scaling.

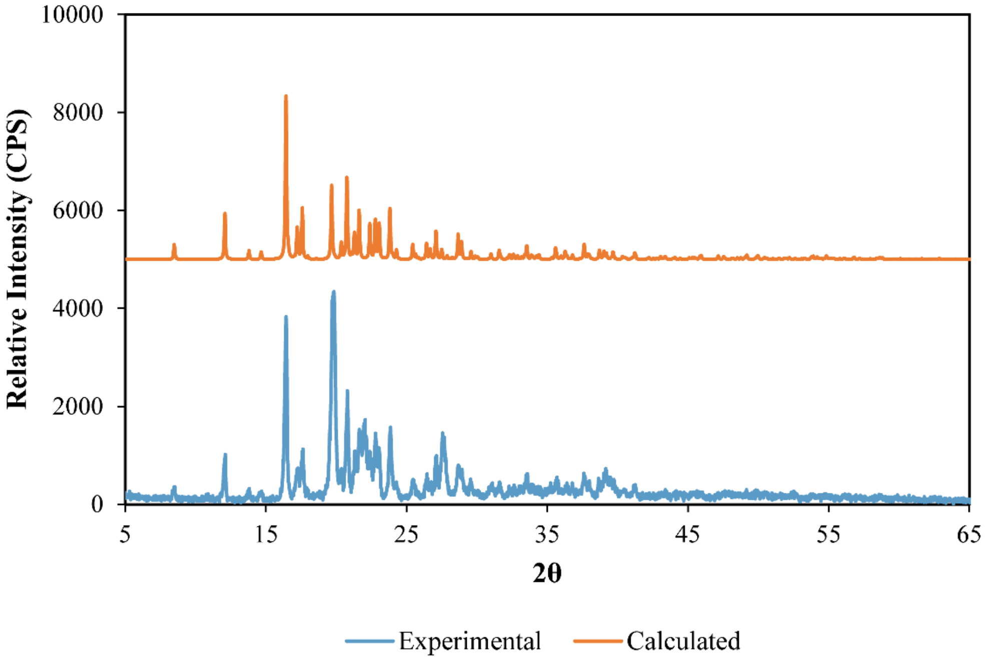

Supplementary Figure 13. Experimental and calculated powder X-ray diffractogram of compound **4** [(2,3,5,6-tetramethylpyrazine)(1,4-cyclohexanedicarboxylic acid)].

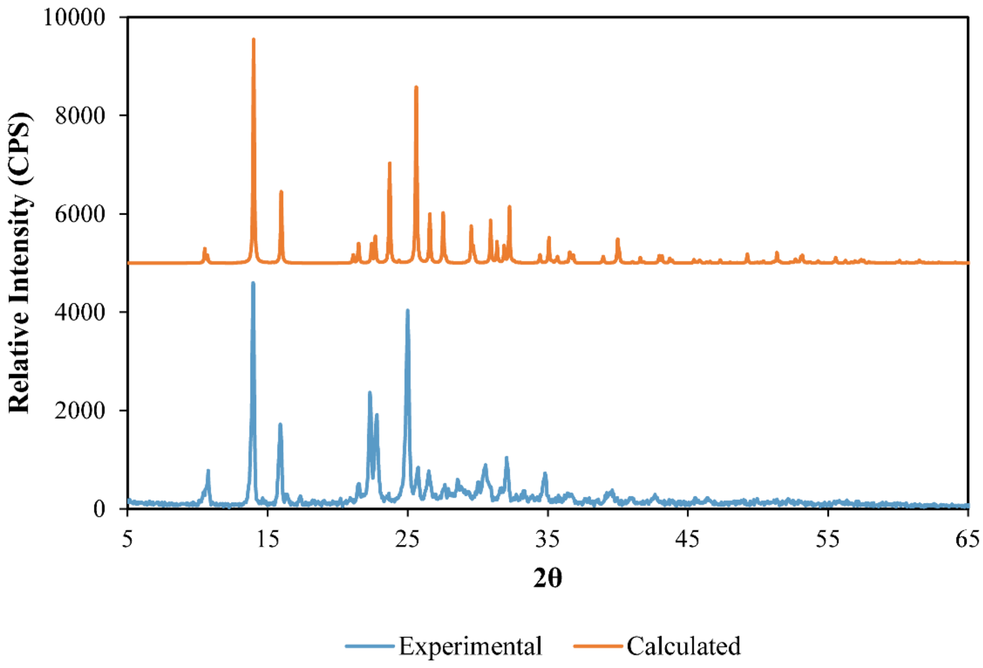

Supplementary Figure 14. Experimental and calculated powder X-ray diffractogram of compound **5** [(2,3,5,6-tetramethylpyrazine)(oxalic acid)].

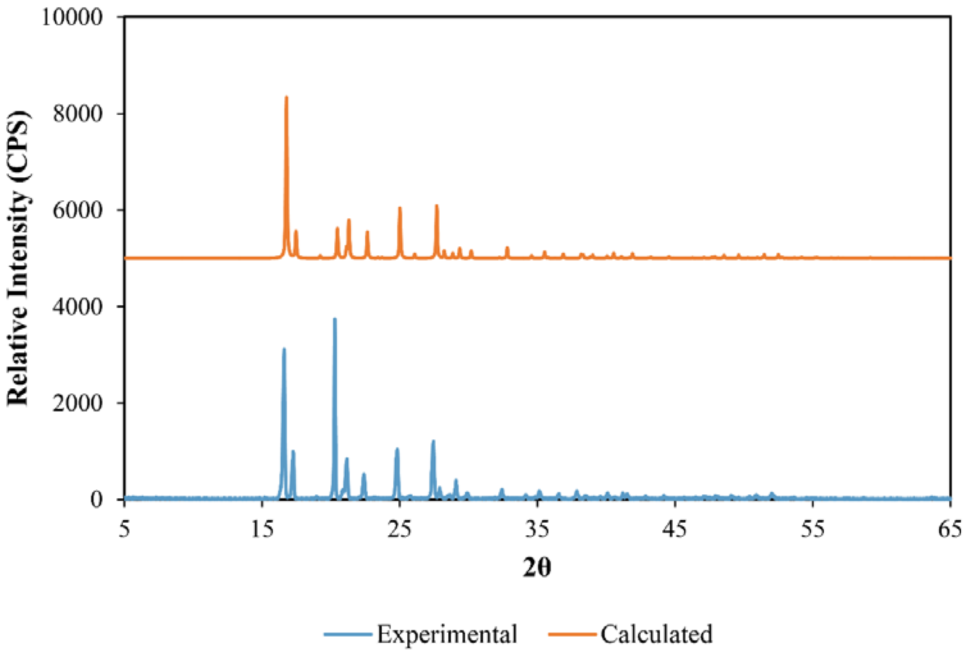

Supplementary Figure 15. Experimental and calculated powder X-ray diffractogram of compound **6** [2,3,5,6-tetramethylpyrazine].

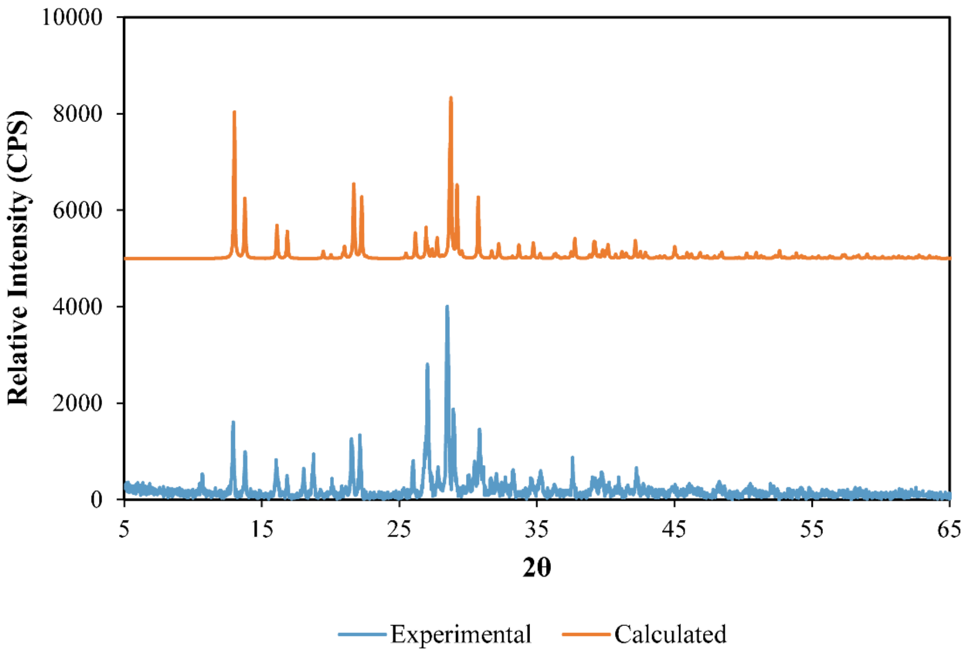

Supplementary Figure 16. Experimental and calculated powder X-ray diffractogram of compound **7** [2,3,5,6-tetramethylpyrazinium hydrochloride].

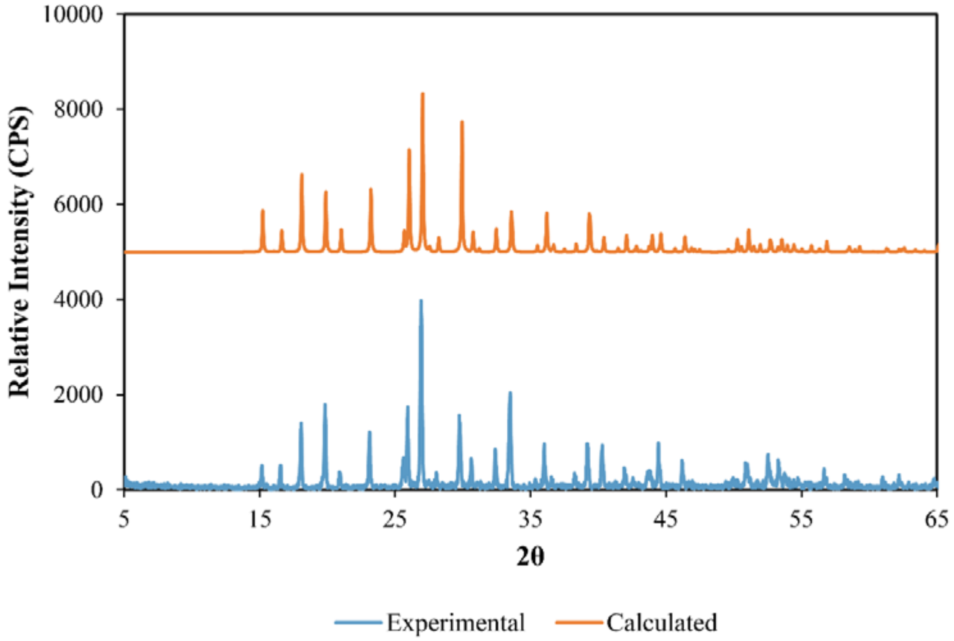

Supplementary Figure 17. Experimental and calculated powder X-ray diffractogram of compound **8** [2,3,5,6-tetramethylpyrazinium hydrobromide].

Supplementary References.

[1] Vold, R.L. & Hoatson, G.L. Effects of jump dynamics on solid state nuclear magnetic resonance line shapes and spin relaxation times. *J. Magn. Reson.* **198**, 57-72 (2009).

[2] ADF2017, SCM, Theoretical Chemistry, Vrije Universiteit, Amsterdam, The Netherlands.
